# FMN riboswitch aptamer symmetry facilitates conformational switching through mutually exclusive coaxial stacking configurations

**DOI:** 10.1016/j.yjsbx.2020.100035

**Published:** 2020-08-06

**Authors:** Haley M. Wilt, Ping Yu, Kemin Tan, Yun-Xing Wang, Jason R. Stagno

**Affiliations:** a Structural Biophysics Laboratory, Center for Cancer Research, National Cancer Institute, Frederick, MD 21702, USA; b Structural Biology Center, X-ray Science Division, Advanced Photon Source, Argonne National Laboratory, 9700 S. Cass Ave., Lemont, IL 60439, USA; c Washington College, Chestertown, Maryland 21620, USA

**Keywords:** RNA structure, Riboswitch, Gene regulation, Structural biology, X-ray crystallography, Flavin mononucleotide

## Abstract

Knowledge of both apo and holo states of riboswitches aid in elucidating the various mechanisms of ligand-induced conformational “switching” that underpin their gene-regulating capabilities. Previous structural studies on the flavin mononucleotide (FMN)-binding aptamer of the FMN riboswitch, however, have revealed minimal conformational changes associated with ligand binding that do not adequately explain the basis for the switching behavior. We have determined a 2.7-Å resolution crystal structure of the ligand-free FMN riboswitch aptamer that is distinct from previously reported structures, particularly in the conformation and orientation of the P1 and P4 helices. The nearly symmetrical tertiary structure provides a mechanism by which one of two pairs of adjacent helices (P3/P4 or P1/P6) undergo collinear stacking in a mutually exclusive manner, in the absence or presence of ligand, respectively. Comparison of these structures suggests the stem-loop that includes P4 and L4 is important for maintaining a global conformational state that, in the absence of ligand, disfavors formation of the P1 regulatory helix. Together, these results provide further insight to the structural basis for conformational switching of the FMN riboswitch.

## 1 Introduction

RNA riboswitches get their names from the conformational “switching” that occurs upon binding of key cellular metabolites, which enables control of transcription, translation, or splicing of the gene(s) with which the riboswitch is associated (Breaker, 2012, Mandal et al., 2003, Serganov and Patel, 2007). They commonly reside in the 5′ untranslated regions of certain prokaryotic mRNAs, and consist of two components: an aptamer domain, and an expression platform. Each aptamer domain specifically recognizes a small-molecule metabolite, the binding of which triggers conformational changes in the aptamer and expression domains, and enables the control of downstream genes (Breaker, 2018, Mandal and Breaker, 2004). Ligand-induced structural changes in the expression platform control the formation of a hairpin structure that terminates transcription prematurely, or a structure that sequesters the ribosome binding site and inhibits translation (Barrick and Breaker, 2007). The flavin mononucleotide (FMN) riboswitch, or *RFN* element, is a widespread and highly conserved RNA structural element present in the eubacterial operons of genes involved in riboflavin biosynthesis and transport (Gelfand et al., 1999, Gusarov et al., 1997, Vitreschak et al., 2002). In response to intracellular concentrations of FMN, a derivative of vitamin B_2_, the FMN riboswitch in most bacteria follows a mechanism of transcription attenuation, whereby ligand binding to the aptamer domain leads to the formation of an intrinsic terminator hairpin followed by a uridine track in the downstream expression platform (Wickiser et al., 2005, Winkler et al., 2002).

Riboswitch structures are highly conserved across species and have been identified as attractive antibiotic targets (Blount and Breaker, 2006, Blount et al., 2007, Serganov et al., 2006, Thore et al., 2008). The development of antibiotics using structure-guided approaches relies on understanding the various conformational states of riboswitch aptamers (Garst et al., 2011, Vicens et al., 2018). This includes not only structures stabilized by ligand binding, but also static or dynamic structures in the absence of ligand, and the regulatory conformational changes they must undergo to actuate genetic control. The high structural similarity amongst riboswitches suggests that their underlying mechanisms share a limited number of common features that are diversified by the highly-selective binding of metabolites that trigger them (Lee et al., 2011, Porter et al., 2014). Detailed knowledge, therefore, of ligand-free (apo) and ligand-bound (holo) states of riboswitches, as well as any stable intermediates, can aid in elucidating those shared mechanisms of conformational “switching,” which regulate many essential metabolic genes (Bailor et al., 2010, Baird and Ferre-D'Amare, 2010, Dussault et al., 2017, Jones and Ferre-D'Amare, 2017, Stagno et al., 2017a, Vicens et al., 2011). Functional RNAs, like riboswitches, particularly in their often more flexible apo states, may adopt multiple conformations capable of chemo-sensing their cellular environment (Liberman and Wedekind, 2012). Such conformations may involve interconversion of binding-competent and binding-incompetent aptamer structures (Stagno et al., 2017b). For riboswitches that are thermodynamically controlled (e.g., adenine riboswitch), equilibrium is achieved when the binding of ligand stabilizes a conformation that is more energetically favorable. For kinetically driven riboswitches (e.g., FMN riboswitch), RNA polymerase encounters the terminator before the aptamer and ligand reach binding equilibrium, in which only a fraction of RNA molecules may exist in the ligand-activated state at the time a gene-regulatory decision is made (Barrick and Breaker, 2007, Batey, 2012, Wickiser et al., 2005, Zhang et al., 2010).

The FMN riboswitch is common amongst many pathogenic bacteria, and, therefore, is of particular interest (Barrick and Breaker, 2007, Blount and Breaker, 2006, Vitreschak et al., 2002). The pivotal and ground-breaking works of Serganov *et al.* and Vicens *et al.* laid the foundation for understanding the FMN riboswitch aptamer on the structural level (Serganov et al., 2009, Vicens et al., 2011). Comparative X-ray crystallography and small-angle X-ray scattering (SAXS) studies showed that the FMN riboswitch is globally folded in the absence of ligand and undergoes minimal conformational changes upon ligand binding (Baird and Ferre-D'Amare, 2010, Vicens et al., 2011). Ligand-induced stabilization of the aptamer’s P1 helix results in the formation of an intrinsic terminator stem in the expression platform (Winkler et al., 2002). This is facilitated in part by the intercalation of FMN’s isoalloxazine ring system between the bases of A48 and A85, thereby bridging the gap between helices P1 and P6 (Serganov et al., 2009, Vicens et al., 2011). However, reported structures of the aptamer provide only a partial explanation for the ligand-induced conformational switch, as the structure and orientation of the regulatory P1 helix is virtually identical in the apo and holo states. The observed coaxial alignment of P1 and P6 in the previously reported apo structure does not explain why the formation of P1 is significantly less favorable without the single-strand base-stacking that FMN affords. To further investigate the underlying conformational switching mechanism of the FMN riboswitch, we proposed that the aptamer adopts an alternate conformation that is more favorable in the absence of FMN. Here, we report a 2.7-Å resolution crystal structure of an apo conformation of the FMN riboswitch aptamer (PDB 6WJR), referred to throughout as “apo-6WJR.” The structure is distinct from previously reported apo and holo structures, and provides a more complete structural basis for conformational switching that involves mutually exclusive coaxial stacking configurations, exploited by the aptamer’s rare symmetrical fold.

## 2 Materials and methods

### 2.1 RNA preparation

The 112-nt RNA sequence for the *F. nucleatum* FMN riboswitch aptamer is the same as described in (Serganov et al., 2009). The linear DNA template (147 base pairs), containing the T7 RNA polymerase promoter, was synthesized (Integrated DNA Technologies), and amplified by PCR using the following primers: 5′ – TCTGATTCAGCTAGTCCATAATACGACTCACTATAGG (forward), and 5′ – mGmAATCTTCTCTCATCCAGACTCTACTG (reverse). The reverse primer contained 2′-O-methylated nucleotides (mX) at the 5′-end of the coding strand to avoid non-templated addition to RNA transcripts (Kao et al., 1999). The RNA was transcribed *in vitro* using T7 RNA polymerase for 4 h at 37 °C, and purified by native polyacrylamide gel electrophoresis. Pure RNA was eluted from the gel at 4 °C in a buffer containing 50 mM sodium acetate, pH 5.3, 2 mM EDTA. The following day, the sample was filtered, buffer exchanged into RNA buffer: 10 mM HEPES, pH 7.5, 100 mM KCl, 1 mM MgCl_2_, 0.1 mM EDTA, and concentrated to ~12 g/L using an Amicon® Ultra-15 3 kDa cutoff centrifugal filter unit (Millipore). Three microliters of SUPERase•In™ RNase Inhibitor (Thermo Fisher Scientific) were added to the pure, concentrated sample, and stored at −80 °C until use.

### 2.2 Crystallization

RNA was thawed on ice and centrifuged at >15,000*g* to remove particulate matter. For the apo-6WJS RNA sample, the MgCl_2_ concentration was adjusted to 5 mM prior to centrifugation. Sitting-drop, vapor-diffusion crystallization experiments were performed using a Crystal Gryphon (Art Robbins Instruments) and incubated at 20 °C in a RockImager (Formulatrix). Apo-6WJR crystals grew in crystallization conditions containing 0.1 M sodium acetate, pH 4.6, 0.2 M ammonium sulfate, and 26% PEG 3350. Crystals were cryoprotected in buffer containing 0.1 M sodium acetate, pH 4.6, 0.2 M ammonium sulfate, and 30% PEG 3350, and 10% glycerol. Crystals of apo-6WJS were obtained from crystallization buffer containing 0.1 M SPG (succinic acid, sodium phosphate monobasic monohydrate, glycine; 2:7:7 ratio), pH 9.0, and 25% PEG 1500, and were cryoprotected in the same crystallization buffer supplemented with 20% glycerol. Crystals were flash-frozen in liquid nitrogen. Data were collected at the Advanced Photon Source (APS), Argonne National Lab, beamlines 23-ID (apo-6WJR) and 19-ID (apo-6WJS).

### 2.3 Structure determination

Crystal data were processed using *XDS* (Kabsch, 2010). The structure of apo-6WJR was determined by *PHASER* (McCoy et al., 2007) molecular replacement using the *PHENIX* software suite (Liebschner et al., 2019), using holo-3F2Q (Serganov et al., 2009) as a search model. The structure of apo-6WJS was determined in the same manner using apo-6WJR as the search model. Manual model-building was performed in *Coot* (v. 0.8.9.2) (Emsley et al., 2010). Structure refinement was performed using *PHENIX* (v. 1.18rc5-3822). The refinement strategy of apo-6WJR included energy minimization of atomic coordinates, individual atomic displacement factors (ADPs) with auto-generated translation/libation/screw (TLS) parameters, and automatic optimization of the X-ray/ADP weighting factor. Interestingly, however, sizable (3 to 4 sigma) peaks of uninterpretable positive difference electron density were observed near the P1 termini. We suspect these peaks are partial occupancy of large molecules from the crystallization buffer or short fragments of degraded RNA. However, the difference density could not be modelled with any degree of confidence, and was left unaccounted for in the final model. The refinement strategy of apo-6WJS included deformable elastic network (DEN) refinement (Schroder et al., 2010) with automatic optimization of DEN parameters, group ADPs with auto-generated TLS parameters, and automatic optimization of X-ray/stereochemistry and X-ray/ADP weighting factors. Crystal data and refinement statistics can be found in Table S2. All figures were generated using The PyMOL Molecular Graphics System, Version 2.3.2, Schrodinger, LLC.

## 3 Results

### 3.1 Differences between apo and holo structures of the FMN riboswitch aptamer

**Fig. 1:**
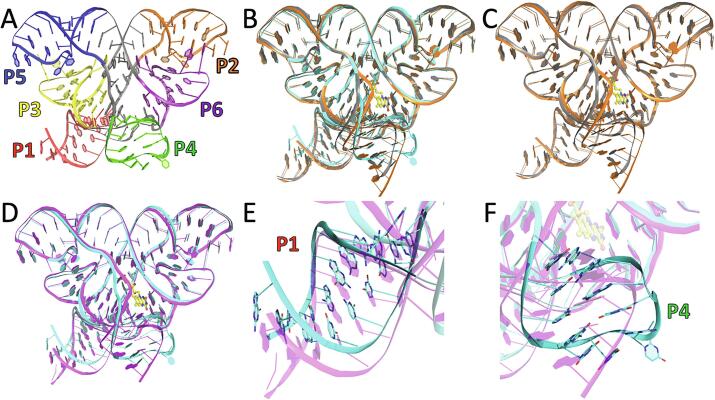
Structural differences between apo and holo aptamer conformations. (A) Cartoon of the butterfly-shaped tertiary structure of apo-6WJR, colored by duplex regions (P1-P6), with connecting segments comprising the six-way junction colored in gray. A map of the secondary structure is shown in Fig. S1. Superimposed structures: (B) apo-6WJR (cyan), apo-2YIF (gray), and holo-2YIE (orange); (C) apo-2YIF (gray) and holo-2YIE (orange); (D) apo-6WJR (cyan) and holo-3F2Q (magenta). (E-F) Expanded views of the structural alignment shown in (D) for the opposing helical domains, P1 (E) and P4 (F), which exhibit the largest structural deviations.

The FMN riboswitch aptamer from *F. nucleatum* exhibits a “butterfly-like” fold, whose six helical domains form nearly symmetrical “wings” with a central junction area that includes the FMN binding site (Fig. 1A) (Serganov et al., 2009, Vicens et al., 2011). Previously reported apo and holo structures that were used here for structural comparison with apo-6WJR include: the FMN-bound structure, “holo-3F2Q” (PDB ID: 3F2Q, 2.95 Å resolution) (Serganov et al., 2009), which shared the same RNA construct as this work, and the apo and bound structures, “apo-2YIF” (PDB ID: 2YIF, 3.30 Å resolution) and “holo-2YIE” (PDB ID: 2YIE, 2.94 Å resolution), which were acquired using two independently synthesized and annealed strands engineered to promote crystal packing (Vicens et al., 2011). The backbone atoms of all residues except 1–9, 47–60, and 104–113 were used for structure alignment. All four structures are nearly identical with respect to the two “peripheral” domains, P2/P6 and P3/P5, which provide a rigid aptamer scaffold (Fig. 1). The differences between apo-2YIF and holo-2YIE (all-residue RMSD of 1.21 Å) are primarily localized to the junction regions surrounding the ligand binding site, but also includes a translational shift in P4 by <3 Å (Fig. 1C) (Vicens et al., 2011). The structure of apo-6WJR reveals greater differences overall, with RMSD values of 2.59, 2.06, and 2.77 Å, when compared with holo-3F2Q, apo-2YIF, or holo-2YIE, respectively (Fig. 1, Table S1). The most significant differences, however, reside in the structures and orientations of helices P1 and P4 (Fig. 1E and 1F, Table S1).

**Fig. 2:**
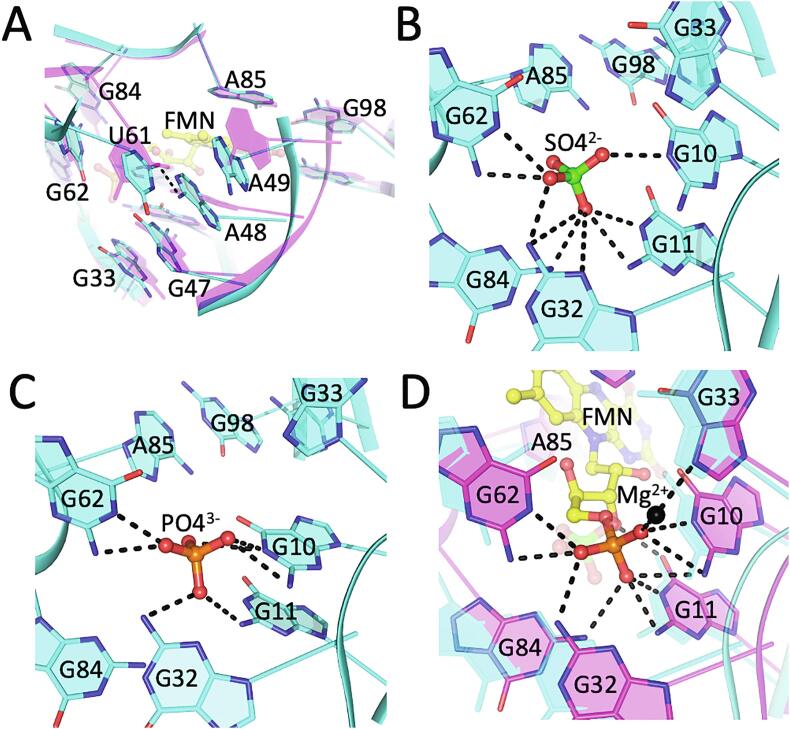
Structure of the FMN-binding pocket. (A) Conformation of the purine-rich region surrounding the isoalloxazine chromophore, as observed in the structures of apo-6WJR (cyan) and holo-3F2Q (magenta). In the absence of FMN, junction residues U61, A48, and A49 reposition to form base stacking interactions (G47, A48, and A49), stabilized by non-WC base-pairing between A48 and U61, (B-C) FMN-phosphate-coordinating side of the binding site depicting hydrogen-bond interactions coordinating a sulfate (B) or phosphate (C) ion, as observed in the respective crystal structures of apo-6WJR and apo-6WJS, which mimic the phosphate moiety of FMN in the holo structure. (D) Structure of the FMN-binding pocket of holo-3F2Q (magenta), showing the coordination of the FMN ligand and a Mg^2+^ ion (black sphere). The superimposed structure of apo-6WJR (cyan) is included for reference. FMN is depicted as yellow ball-and-stick. Hydrogen-bond and ion-coordinating interactions are drawn as a black dashed lines.

Residues comprising the FMN-binding pocket exhibit minor changes upon ligand binding. However, local shifts of these crucial junctional residues led to significant rearrangements of the overall coaxial stacking configurations. Specifically, P6 residues (A85, G98, A99), located on the isoalloxazine-side of the FMN binding pocket, remain relatively fixed, whereas junction residues G47, A48, A49 bridge and conduct base stacking between helices P3 and P4. This is further stabilized by a non-Watson-Crick base pair between U61 and A48 (Fig. 2A and S1). The positions of the guanine residues (G10, G11, G32, G62, G84) that coordinate the FMN phosphate moiety are relatively unchanged. This is due, in part, to a sulfate ion that mimics the FMN phosphate with a similar coordination sphere (Fig. 2B and 2D). We determined another crystal structure of the same RNA (apo-6WJS) under different crystallization conditions containing phosphate instead of sulfate (Fig. 2C). This structure is nearly identical to apo-6WJR, and revealed similar hydrogen-bond interactions for the tetrahedral ion. In the structures of holo-2YIE and holo-3F2Q, the FMN phosphate is further coordinated by a Mg^2+^ ion, which in turn is coordinated by N7 of G33. The Mg^2+^ concentrations in the crystallization buffer for those two structures were 0.32 M and 0.1 M, respectively. In the cases of apo-6WJR and apo-6WJS, which were crystallized at low concentrations of 0.5 mM and 2.5 mM MgCl_2_, respectively, and both containing a tetrahedral ion at the position of the FMN phosphate moiety, no ordered Mg^2+^ was observed. This indicates that the presence of the ordered Mg^2+^ observed in the holo structures may not be dependent on the presence of FMN alone, but also on [Mg^2+^]. Indeed, Serganov *et al.* demonstrated the dependence of FMN binding on [Mg^2+^] (or similar divalent cation) (Serganov et al., 2009).

Nevertheless, the positions of G10 and G11 in apo-6WJR are artificially imposed by the tetrahedral ion, as they reside in the switching sequence (residues 6–11) that forms a competing stem-loop in the apo form of the full-length riboswitch (Fig. S1). Several other crystal forms were obtained under conditions absent of tetrahedral anions. However, all such crystals diffracted very poorly (<10 Å resolution), most likely owing to increased flexibility of J1/2 and instability of P1. The presence of a tetrahedral anion, and the absence of the expression platform, therefore, enables formation of the P1 helix in apo-6WJR, albeit less stable than its ligand-bound form. Partial instability of P1 in apo-6WJR is reflected in the electron density map and significantly elevated B-factors, both of which progressively worsen toward the base of the stem (Figs. S2 and S3). Electron density for the terminal residue, C112, was not observed.

**Fig. 3:**
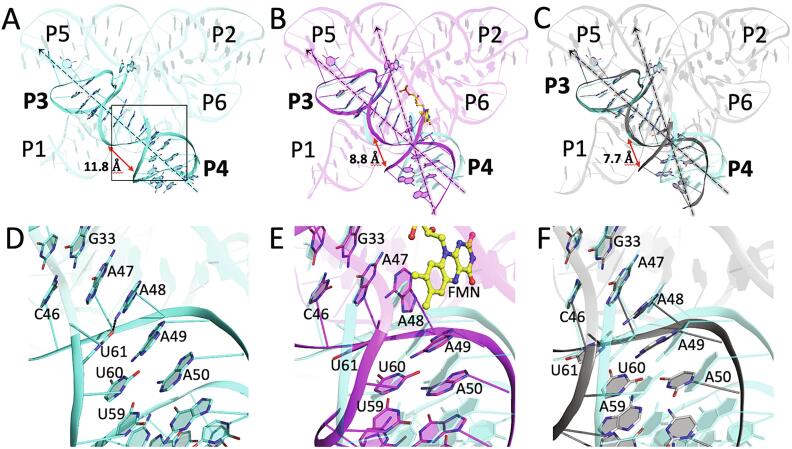
Coaxial alignment of P3 and P4 in the absence of ligand. (A-C) Cartoon depicting the P3 and P4 helices as observed in (A) apo-6WJR (cyan), (B) holo-3F2Q (magenta), and (C) apo-2YIF (gray). Approximate helical axes (depicted as dotted lines) for P3 and P4 are aligned in apo-6WJR (A), and misaligned (kinked) in holo-3F2Q (B) and apo-2YIF (C), which is further illustrated in the reduction of the major groove (red arrows) formed between the two helices, taken as the distance between the phosphorus atoms of residues 46 and 58. (D-F) Expanded view of the P3/P4 helical junction in (A), (B), and (C), respectively, whose area is indicated by the black box in (A). The FMN ligand is shown as yellow ball-and-stick.

The largest deviations in apo-6WJR from previously reported structures are observed in the “radial” domains, P1 and P4, which oppose one another across the aptamer’s pseudo-two-fold axis of internal symmetry (Fig. 1D-1F). Of note, loop L4 is absent in apo-2YIF and holo-2YIE, due to the use of two independent RNA strands, or unobserved in holo-3F2Q (Serganov et al., 2009, Vicens et al., 2011). In both previous studies, however, the same crystal packing interface was observed, in which the short, duplexed region of P4 exhibits helical stacking with P5 of a neighboring symmetry molecule (Fig. S4). This interaction may be an artifact of crystallization. From the existing models, it is not clear how the missing three residues (54–56) could be modeled to form a tetraloop. In addition, the incomplete P4 helix is kinked relative to P3, revealing a very narrow and unfavorable major groove (apo-2YIF: 7.7 Å, holo-3F2Q: 8.8 Å, apo-6WJR: 11.8 Å) for an otherwise unconstrained helix (Fig. 3A-3C) (Serganov et al., 2009, Vicens et al., 2011).

**Fig. 4:**
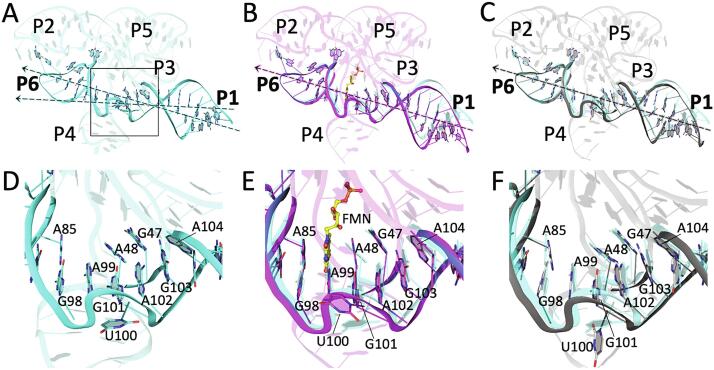
FMN stabilizes helix P1 by inducing coaxial alignment with helix P6. (A-C) Cartoon depicting the P1 and P6 helices as observed in (A) apo-6WJR (cyan), (B) holo-3F2Q (magenta), and (C) apo-2YIF (gray). The orientation relative to Fig. 3 is ~180° around the vertical axis. Approximate helical axes (depicted as dotted lines) for P1 and P6 are aligned in holo-3F2Q (B) and apo-2YIF (C), and misaligned in apo-6WJR (A). (D-F) Expanded view of the P1/P6 helical junction in (A), (B), and (C), respectively, whose area is indicated by the black box in (A). (D) The absence of FMN in apo-6WJR creates a gap and kink between the P1 and P6 helices. Junction residues, A48, A49, and A104 reorient to propagate stacking between P3 and P4 helices, resulting in the partial instability of P1. (E) In the presence of ligand, the continuity and alignment of P1 and P6 is facilitated by the isoalloxazine ring of FMN (yellow ball-and-stick) through base-stacking with A85 and A48, thereby stabilizing P1. (F) In apo-2YIF, A48, A49, and A104 exhibit changes similar to those in apo-6WJR. However, the alignment of P1 and P6 is reminiscent of a bound-like conformation.

**Fig. 5:**
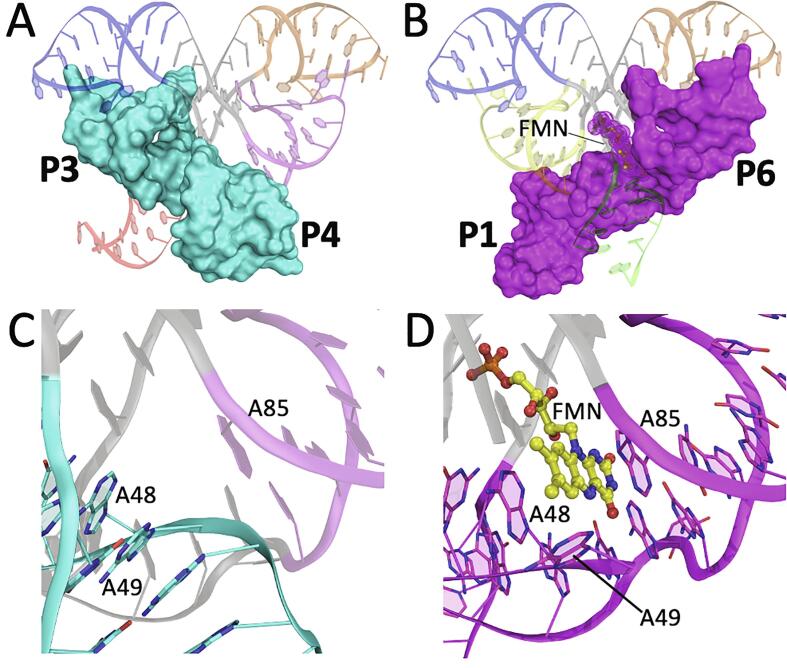
Conformational switching of the FMN riboswitch aptamer through mutually exclusive coaxial stacking. Cartoons of apo-6WJR (A) and holo-3F2Q (B), showing the coaxial alignment of P3/P4 (cyan) or P1/P6 with FMN (magenta), respectively, depicted as molecular surfaces. (C-D) Expanded view of the coaxial junction, as observed in apo-6WJR (C) and holo-3F2Q (D), that enables conformational switching of the aptamer through mutually exclusive coaxial stacking interactions that exist in the presence or absence of ligand.

Importantly, P4 is adjacent to the FMN-binding site and may play a role in modulating P1 helix stability. In the apo conformation, A48 forms stacking interactions with A49. Thus, J3/4 aligns helices P3 and P4 (Fig. 3A). Although similar base-stacking is observed between A48 and A49 in apo-2YIF, the P3 and P4 helices are misaligned, or kinked, as they are in holo-2YIE and holo-3F2Q (Fig. 3B and 3C, Fig. S5). Therefore, although the structure of apo-2YIF demonstrates the role of J3/4 in locally maintaining an “open” state productive for ligand binding, the energetic basis for the structural switching behavior is not apparent. Our results suggest that P3/P4 stacking is more favorable in the absence of FMN, resulting in the loss of P1 stability and liberation of the switching sequence to the competing terminator stem. This is explained through the productive coaxial alignment of P3/P4 and misalignment of P1/P6 (Fig. 4A). Conversely, when FMN is bound, its isoalloxazine ring stacks with A48 and A85, thereby switching coaxial alignment to P1/P6 (Fig. 4B) (Serganov et al., 2009, Vicens et al., 2011). At high FMN concentrations, the misalignment of P3/P4 may lead to instability of L3 and L4, as indicated in prior SHAPE mapping experiments (Vicens et al., 2011). The junction region of the aptamer, therefore, functions like a railway switch-track. Despite involving minimal changes in or near the binding pocket, FMN binding switches the direction of productive base stacking, not only *within* the junction, but more importantly *across* the junction, from collinearity of P3/P4 (apo) to collinearity of P1/P6 (holo) (Fig. 5). The crisscrossing of mutually exclusive aligned helices is facilitated by the symmetrical architecture of the aptamer.

## 4 Discussion

Previously reported structures of the FMN riboswitch aptamer reveal minimal differences between the apo and holo conformations, primarily localized to the ligand-binding pocket (Serganov et al., 2009, Vicens et al., 2011). Given the size and complexity of this aptamer, and its unique internal pseudo-symmetry, such minimal local changes without affecting the conformations and orientations of other domains do not provide an adequate molecular basis for the aptamer’s ligand-triggered switching behavior. It is possible that the same crystal-packing interface shared by the apo and holo structures had constrained the conformation of the otherwise more different conformation of the apo form. This is, of course, difficult to fully assess without the expression platform, which forms a competing stem-loop in the absence of ligand and precludes P1 formation. As such, productive apo conformations of the aptamer would be expected to disfavor P1 helix formation and stability, so as not to compete with the terminator in the absence of ligand (Aboul-ela et al., 2015, Batey, 2012). We have identified a ligand-free structure of the aptamer that provides further insight into the conformational switching mechanism that enables genetic control by this riboswitch. Apo-6WJR shows major differences in P1 and P4 compared to the holo conformation, which strongly indicates regulation through the formation of one of two mutually exclusive coaxial stacking configurations.

In the absence of FMN, the helical structures of P3 and P4 are aligned, the stacking continuity of which is facilitated by junction residues G47, A48, and U61. This results in misalignment of P1 and P6, destabilizing P1, and liberating the switching sequence that includes J1/2. In apo-6WJR, the otherwise mobile J1/2 is stabilized by a sulfate (or phosphate) ion that mimics the phosphate moiety of the FMN ligand. This, and the absence of the expression domain, explains the existence of P1, although the weaker electron density in this region indicates that it is not stably formed as in the holo conformation. By contrast, the structure of apo-2YIF, which contains no tetrahedral anion, has a fully formed P1 helix that is aligned with P6 (Vicens et al., 2011). The “bound-like” conformation could be due to the engineered or absent residues in L4 that ultimately affect P4 structure and alignment with P3, the stabilizing crystal packing interface involving P1, and the high [Mg^2+^] present in the crystallization buffer (0.32 M), all of which may further stabilize the structure in the crystallographically preferred conformation. The ring structure of FMN induces coaxial stacking of P1/P6, thereby stabilizing the P1 helix and, in the context of the full-length riboswitch, precludes formation of the competing terminator stem (Serganov et al., 2009, Vicens et al., 2011). The phosphate moiety of FMN, accompanied by a coordinated Mg^2+^ (Fig. 2D), serves primarily as a stabilizer and ligand-binding enhancer. In the holo structures of the aptamer with the FMN analogues, riboflavin and roseoflavin, both of which lack the phosphate moiety, the binding pocket reveals minimal accommodating changes (Serganov et al., 2009). This is consistent with the sulfate (or phosphate) ion coordination observed in apo-6WJR, which forms similar interactions with J1/2 as in the holo structures, but is insufficient to fully form and align P1 with P6.

Helical stacking has been identified as an important structural feature related to gene regulation in other riboswitches (Barrick and Breaker, 2007, Li et al., 2019, Liberman and Wedekind, 2012, Liberman et al., 2013, Serganov et al., 2008, Stagno et al., 2017a, Stagno et al., 2017b). For kinetically driven riboswitches, such as the FMN riboswitch, a short time-window exists for the aptamer to sample the cellular environment through conformational selection. Therefore, regulatory changes in aptamer structure must be small enough to bind ligand quickly and productively, yet significant enough to energetically favor one competing structure over another. The pseudo-symmetrical fold of the FMN riboswitch aptamer provides a strategy by which a riboswitch can achieve gene expression control by coupling local, junctional changes triggered by ligand binding with global energetic differentials between competing coaxial stacking arrangements. Such a mechanism, which is likely also employed by other riboswitches, would not necessarily require internal aptamer symmetry, but can be effectively executed by a multi-helical junction.

Elucidating the conformation-switching mechanisms of gene regulation by riboswitches could play a key role in the design of novel antimicrobials. Targeting ligand-bound structures of aptamers for the development of antimicrobials often involves competitive binding of ligand analogs (Vicens et al., 2018). However, since these ligands are often primary cellular metabolites, highly similar analogs are more likely to have deleterious off-target effects on host-cell metabolism (Mulhbacher et al., 2010). To that end, detailed structural knowledge of both apo and holo aptamer conformations, or their intermediates, may aid in the design of small molecules capable of trapping the aptamer in either “active” (structurally similar to the ligand-bound conformation) or “inactive” (structurally different from the ligand-bound conformation) states. Such compounds that target RNA conformational states other than the bound form avoid direct competition with the cognate ligand in the host cell, which usually exhibits the highest affinity and are in high abundance. Instead, small molecules that selectively stabilize certain aptamer conformations that have been termed “high-energy states” (e.g., through altered coaxial alignment in this case), may provide an alternative or complementary approach to antimicrobial design.

## 5 Accession numbers

The coordinates and structure factors for apo-6WJR and apo-6WJS were deposited in the Protein Data Bank under accession codes, 6WJR and 6WJS, respectively.

## CRediT authorship contribution statement

**Haley M. Wilt:** Investigation, Visualization, Writing - original draft, Writing - review & editing. **Ping Yu:** Resources. **Kemin Tan:** Resources, Writing - review & editing. **Yun-Xing Wang:** Conceptualization, Supervision. **Jason R. Stagno:** Conceptualization, Formal analysis, Investigation, Visualization, Writing - original draft, Writing - review & editing, Supervision.

## Declaration of Competing Interest

The authors declare that they have no known competing financial interests or personal relationships that could have appeared to influence the work reported in this paper.
