# Supplementary material for: FMN riboswitch aptamer symmetry facilitates conformational switching through mutually exclusive coaxial stacking configurations
Source: J Struct Biol X. 2020 Aug 6;4:100035. doi: 10.1016/j.yjsbx.2020.100035 (PMC7573352; doi:10.1016/j.yjsbx.2020.100035)
Supplement: Supplementary data 5 [file mmc5.docx]

**Supplementary data**

**
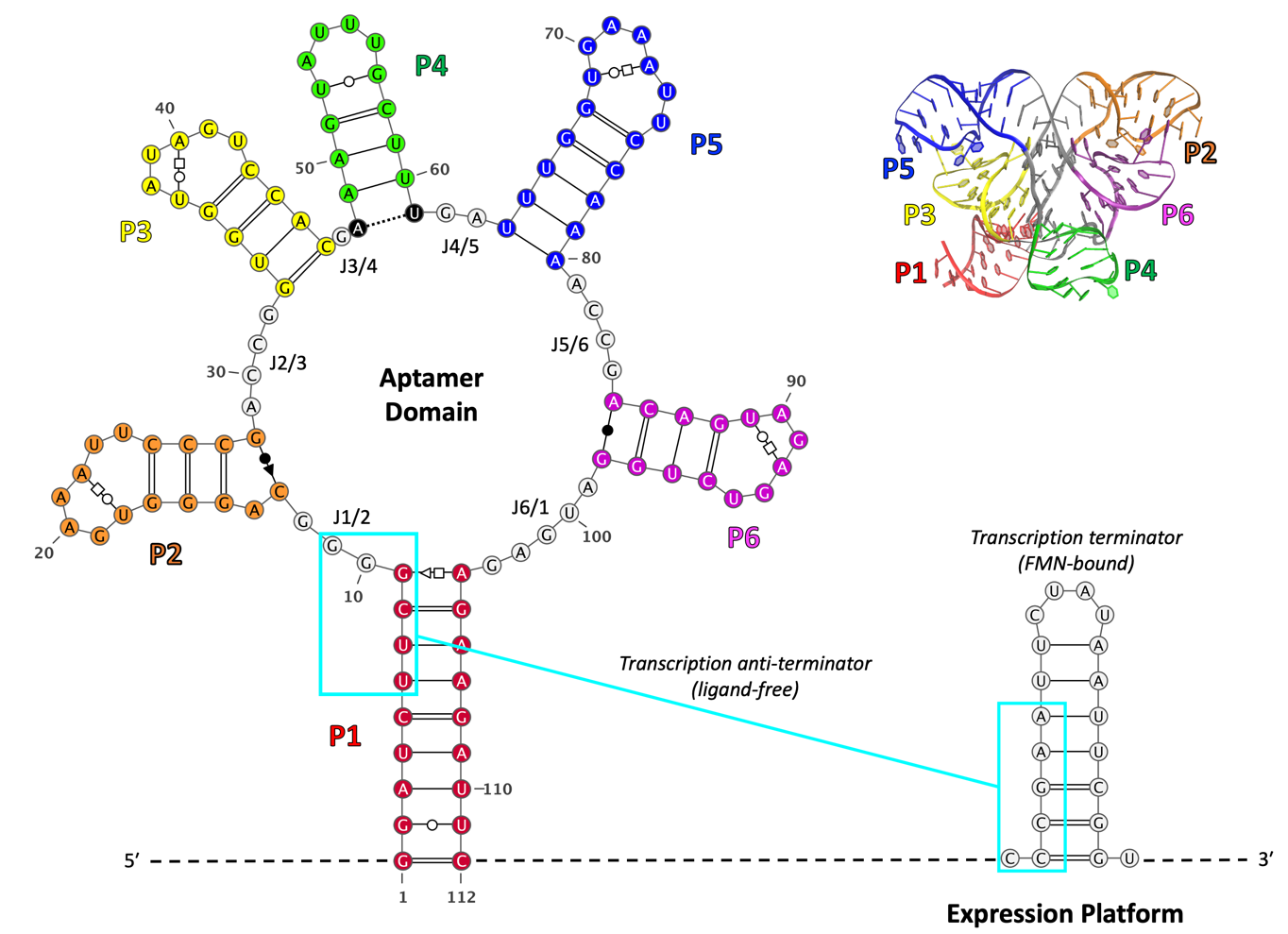
**

**Figure S1. Secondary structure map of the FMN riboswitch.**

A simplified representation of the full-length FMN riboswitch showing the aptamer (FMN-sensing) domain and expression platform. Aptamer residues are color-coded by individual domains (P1-P6). The interaction that is formed between A48 and U61 (black) in apo-6WJR, due to the absence of FMN, is indicated with a dashed line. Base-pairing is annotated using Leontis-Westhoff nomenclature (Leontis and Westhof, 2001), derived using 3DNA-DSSR (Lu and Olson, 2003), and the map was generated using VARNA (Darty et al., 2009). For clarity, long-range base-pairing interactions are not shown. Binding of FMN at the central junction region of the aptamer domain stabilizes the P1 helix, which contains the switching sequence (cyan box), and leads to the formation of the terminator stem in the expression platform (OFF), as shown. In the absence of FMN, destabilization of P1 liberates the switching sequence to form an anti-terminator stem (ON).

**
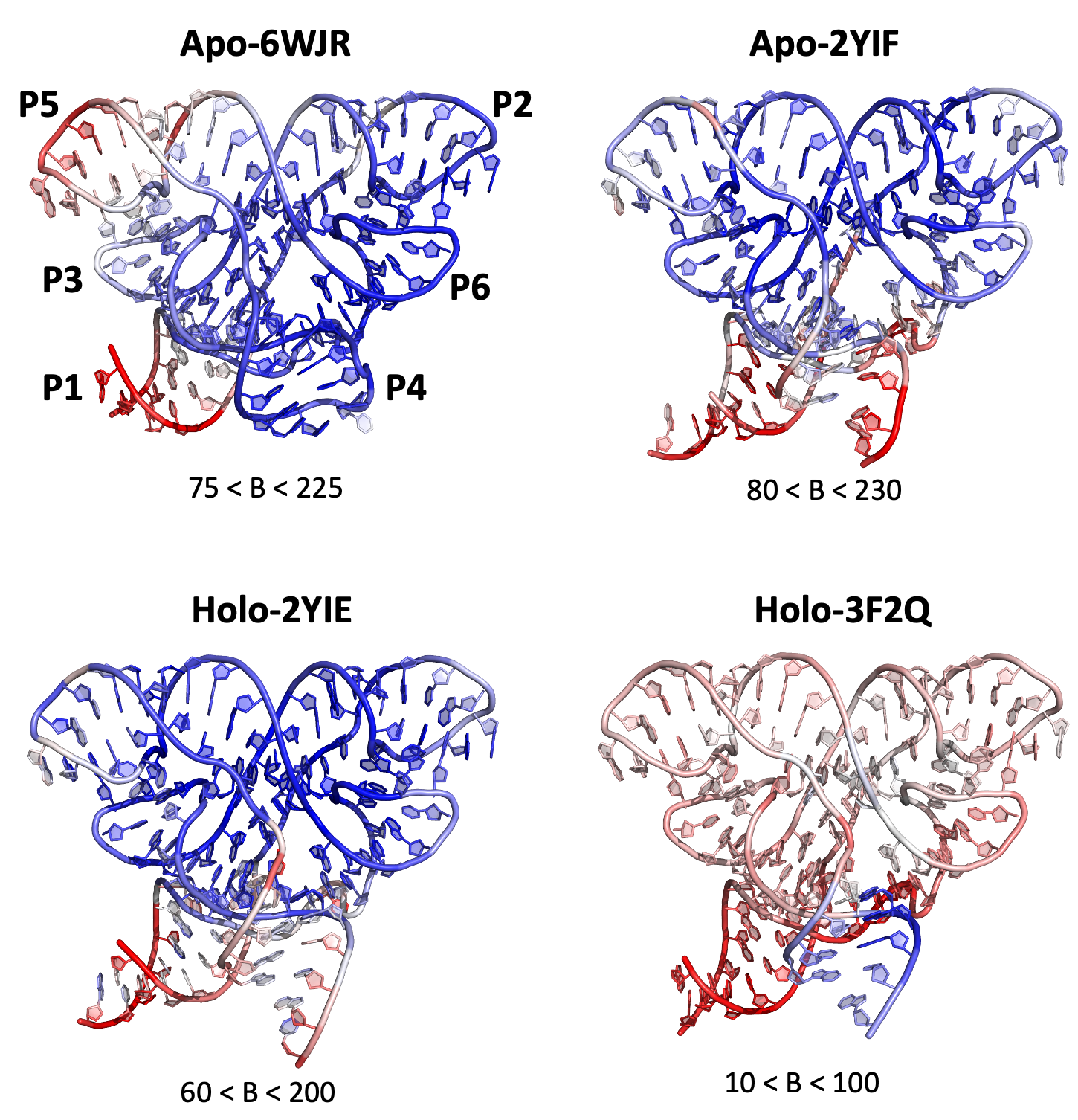
**

**Figure S2. Average B-factor per residue.**

The structures of apo-6WJR, apo-2YIF, holo-2YIE, and holo-3F2Q, colored with respect to average B-factor per residue. The upper and lower limits for the B-factors (Å^2^) were set according to the approximate maximum and minimum isotropic B-factors for each structure, respectively.

**
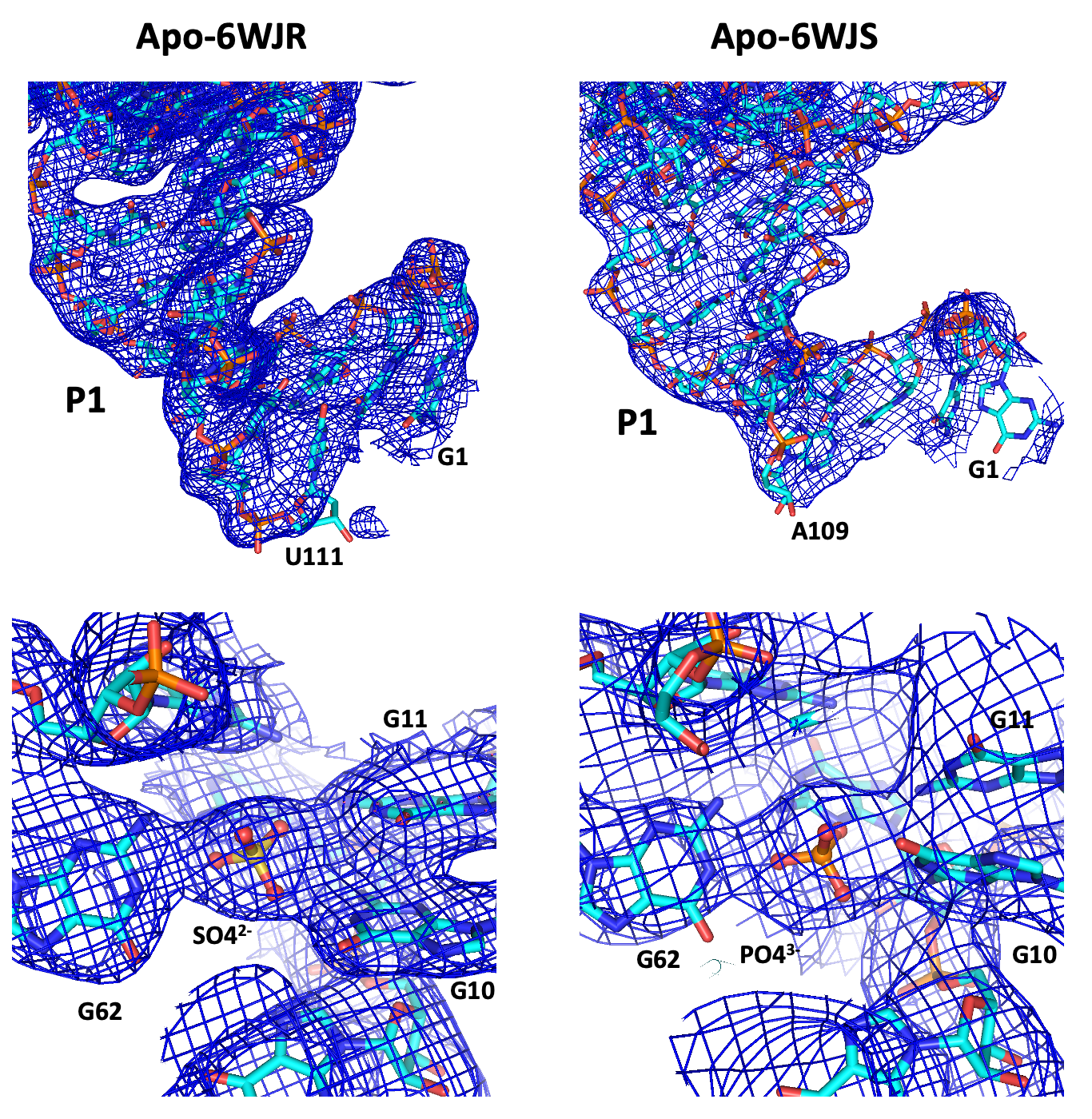
**

**Figure S3. Electron density maps.**

2Fo-Fc electron density maps, contoured at 1 sigma, for the structures of apo-6WJR (left) and apo-6WJS (right).

**
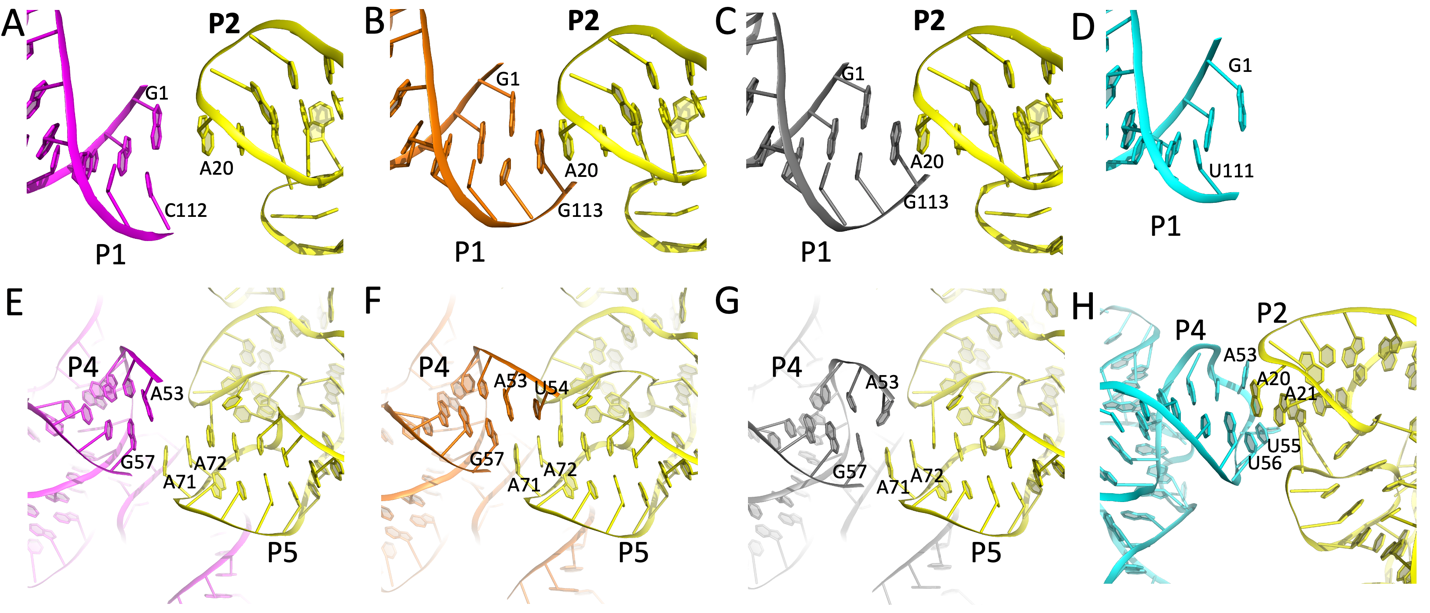
**

**Figure S4. Crystal contacts involving P1 or P4 helices.**

Cartoon representations showing the crystal lattice contacts involving P1 (**A-D**) or P4 **(E-H**), as observed in the structures of (**A, E**) holo-3F2Q (magenta), (**B, F**) holo-2YIE (orange), (**C, G**) apo-2YIF (dark gray), and (**D, H**) apo-6WJR (cyan). Symmetry related molecules for each structure are colored in light gray. In holo-3F2Q (**A**), holo-2YIE (**B**), and apo-2YIF (**C**), which all have the same crystal space group and unit cell, the P1 helix butts up against P2 of an adjacent molecule. This may explain, in part, why the P1 helix of apo-2YIF exhibits the same structure and alignment as its holo-form counterpart. In apo-6WJR (**D**), the P1 helix is completely exposed to solvent and its reduced stability is reflected in the fraying of strands, poor electron density, and elevated B-factors for terminal residues (electron density for terminal residue C112 was uninterpretable and excluded from the model). P4 of holo-3F2Q (**E**), holo-2YIE (**F**), and apo-2YIF (**G**) forms lattice contacts with P5 of an adjacent molecule; although, certain residues in L4 are absent/unobserved in these structures. In apo-6WJR, a different crystal contact is observed for L4, whose tetraloop forms interlocking base-pairs with the tetraloop of P2. For holo-2YIE and apo-2YIF, two independent RNA strands were used containing residues 1-54 and 57-113, respectively, thereby excluding a portion of L4. In addition, parts of helix P4/L4 were modified for the purpose of inducing crystal contacts. Holo-3F2Q and apo-6WJR, on the other hand, contain a single RNA strand that is unmodified in this region. However, the structure and orientation of P4 in holo-3F2Q is highly similar to that of holo-2YIE and apo-2YIF, and electron density for L4 residues 54-56 was not observed. Given the atomic positions of holo-3F2Q residues, A53 and G57, it is unclear how a tetraloop could form, which is seemingly precluded by P5 of the neighboring molecule. In either case (holo-3F2Q or holo-2YIE/apo-2YIF), the structure and orientation of P4 may be unreliable.

**
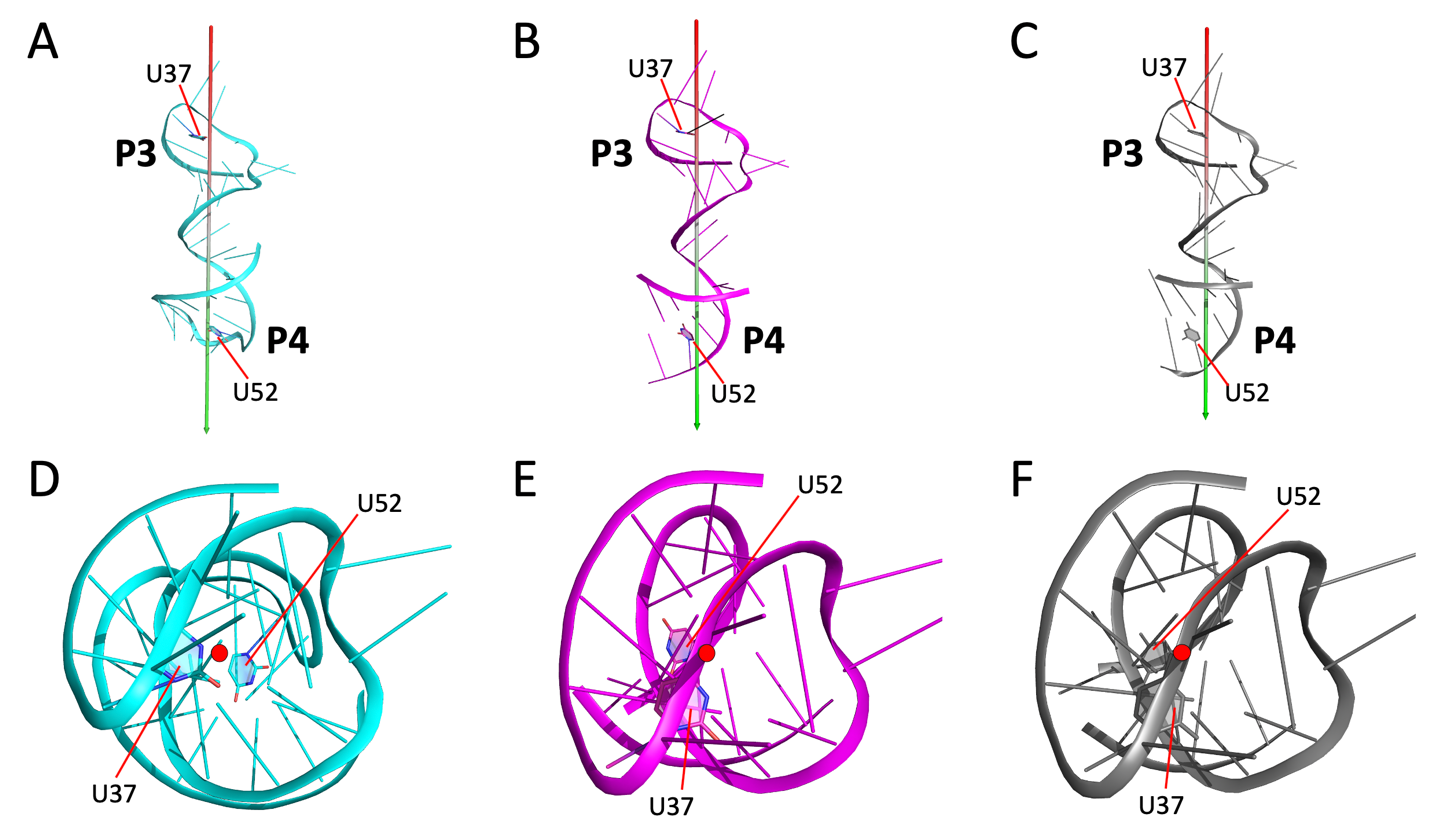
**

**Figure S5. Differences observed in the alignments of P3 and P4 helices.**

Cartoon representation depicting the coaxial alignments of P3 and P4 helices in the structures of apo-6WJR (cyan), holo-3F2Q (magenta), and apo-2YIF (gray). Residues U37 (P3) and U52 (P4) are shown in stick-model for reference. (**A-C**) Sideview showing the P3/P4 alignment in apo-6WJR (**A**), and misalignment in holo-3F2Q (**B**) and apo-2YIF (**C**). (**D-F**) same as (A), (B), and (C), respectively, looking down the helical axis (red circle). The alignment of P3/P4 in apo-6WJR (D) is revealed in the spiraling of residues around the axis from the top of P3 to the bottom of P4.

**Table S1. Root mean square deviations (Å) of various regions of apo-6WJR.**

|  | **holo_3F2Q** | **apo_2YIF** | **holo_2YIE** |
| --- | --- | --- | --- |
| **Residues used for alignment** | 1.28 | 1.04 | 1.08 |
| **All residues** | 2.59 | 2.06 | 2.77 |
| **P1** (1-9, 104-112) | 3.23 | 2.77 | 3.08 |
| **P2** (13-28) | 1.18 | 1.00 | 0.81 |
| **P3** (33-46) | 1.15 | 0.75 | 0.96 |
| **P4** (47-60) | 6.77 | 8.51 | 11.55 |
| **P5** (64-80) | 1.26 | 1.15 | 1.07 |
| **P6** (85-98) | 0.95 | 0.94 | 0.91 |
| **P1-P6** (1-9, 47-48, 99-112) | 2.33 | 2.00 | 2.20 |
| **P3-P4** (33-60) | 4.18 | 3.46 | 5.24 |

The structures, holo_3F2Q, apo_2YIF, and holo_2YIE, were structurally aligned to apo-6WJR using the backbone atoms of all residues except 1-9, 47-60, and 104-112. Alignments were done using *PyMOL* “*align*” with no fitting, and the RMSD values were calculated for backbone atoms using “*rms*_*cur*” function.

**Table S2. Crystallographic data and refinement statistics.**

|  | **Apo-6WJR** | **Apo-6WJS** |
| --- | --- | --- |
| **Data collection** |  |  |
| Beamline | 23-ID, APS | 19-ID, APS |
| Wavelength (Å) | 1.033175 | 1.60747 |
| Resolution range(Å) | 42.14 - 2.70 (2.80 - 2.70) | 41.79 - 3.80 (3.94 - 3.8) |
| Space group | *P* 2_1_ 2 2_1_ | *P* 2_1_ 2 2_1_ |
| Unit-cell parameters (Å, ^o^) | 73.6 36.5 205.5, 90 90 90 | 73.6 36.0 203.0, 90 90 90 |
| Total reflections | 64347 (6787) | 17485 (1800) |
| Unique reflections | 15878 (1309) | 5652 (558) |
| Multiplicity | 4.1 (4.3) | 3.1 (3.2) |
| Completeness (%) | 97.16 (82.43) | 97.43 (99.11) |
| *I*/𝜎(*I*) | 8.26 (0.32) | 5.40 (0.79) |
| Wilson B-factor | 102.34 | 142.19 |
| *R_merge_* | 0.093 (4.542) | 0.150 (1.638) |
| *R*_meas_ | 0.108 (5.162) | 0.181 (1.964) |
| *R*_p.i.m_ | 0.054 (2.417) | 0.100 (1.070) |
| *CC*_1/2_ | 0.997 (0.149) | 0.999 (0.196) |
| *CC** | 0.999 (0.509) | 1.000 (0.573) |
| **Refinement** |  |  |
| Reflections used | 15580 (1309) | 5642 (558) |
| Reflections in test set | 727 (60) | 258 (27) |
| *R*_work_ | 0.2208 (0.4815) | 0.2707 (0.3947) |
| *R*_free_ | 0.2584 (0.5935) | 0.3305 (0.3551) |
| *CC_work_* | 0.859 (0.471) | 0.884 (0.399) |
| *CC_free_* | 0.891 (0.460) | 0.765 (0.524) |
| Number of non-hydrogen atoms | 2390 | 2347 |
| RNA | 2382 | 2342 |
| Ions | 8 | 5 |
| RMS bond lengths (Å) | 0.006 | 0.002 |
| RMS bond angles (^o^) | 1.39 | 0.85 |
| All-atom clash score | 8.1 | 38.64 |
| Average B-factor (Å^2^) | 118.0 | 131.2 |
| RNA | 118.2 | 131.3 |
| Ions | 86.7 | 111.5 |

**References**

Darty, K., Denise, A., Ponty, Y., 2009. VARNA: Interactive drawing and editing of the RNA secondary structure. Bioinformatics 25, 1974-1975.

Leontis, N.B., Westhof, E., 2001. Geometric nomenclature and classification of RNA base pairs. Rna 7, 499-512.

Lu, X.J., Olson, W.K., 2003. 3DNA: a software package for the analysis, rebuilding and visualization of three-dimensional nucleic acid structures. Nucleic Acids Research 31, 5108-5121.
